# Congenital Malaria and Its Associated Factors at Issaka Gazobi Maternity of Niamey in Niger

**DOI:** 10.1155/2020/7802560

**Published:** 2020-10-17

**Authors:** I. Tahirou, M. O. Zara, M. L. Moustapha, M. Kamayé, D. Mahamadou, A. Ibrahim, M. Daou, A. Soumana, M. L. Ibrahim

**Affiliations:** ^1^Faculté de Science de la Santé de l'Université de Niamey, Niger; ^2^Maternité Issaka Gazobi de Niamey, Niger; ^3^Université Cheick Anta Diop de Dakar, Senegal; ^4^Université de Zinder, Niger; ^5^Hôpital National de Niamey, Niger; ^6^Centre de Recherche Médicale et Sanitaire de Niamey, Niger

## Abstract

**Background:**

Congenital malaria is a serious and common infection in tropical Africa. It has multiple consequences on the newborn and the mother.

**Objective:**

The objective of this study is to calculate the prevalence of congenital malaria, describe its clinical signs, and analyze its associated factors. *Methodology*. It is a cross-sectional and prospective study, conducted at Issaka Gazobi Maternity of Niamey, from June 1 to November 30, 2017. The diagnosis was made by microscopy of a thick and thin blood smear of mother, newborn, and umbilical cord.

**Results:**

Two hundred and forty-nine (249) consecutive newborn/mother pairs were included. The prevalence of congenital malaria infection was 26.51% (66/249) with a parasite density of 101 P/*μ*l (SD: 47.3; [80; 320]). The prevalence of congenital malaria disease was 14.06% (35/249) with a parasite density of 108 P/*μ*l (SD: 32.6; [40; 200]. All patients were infected with *Plasmodium falciparum.* 43% (18/35) of neonates had hyperthermia and did not have a sucking reflex, 8.5% (3/35) were anaemic, 11.42% (4/35) had convulsed, 20% (7/35) had a coma, and 45.71% (16/35) had a low birth weight. No deaths were recorded, and only the nonuse of bed nets was significantly associated with congenital malaria (*p* = 0.04).

**Conclusion:**

In Niger, one out of four newborns is infected with *Plasmodium.* Infection can progress to congenital malaria disease. The use of mosquito nets and intermittent preventive treatment would reduce the incidence of congenital malaria.

## 1. Introduction

Congenital malaria is the *in utero* transmission of *Plasmodium* from mother to child during childbirth [1]. Biologically, it is the detection of red blood cells parasitized by the same species of *Plasmodium*, in the mother and the newborn before the seventh day of life. This contamination of the foetus is explained by the fact that the parasite can cross the placental barrier [2], following a decrease in the immunity of the mother [3].

In malaria-endemic areas, congenital malaria is a major public health problem according to the World Health Organization [1]. In tropical Africa, there are 25 million pregnant women each year and 6 million with malaria [4]. 75,000 to 200,000 newborn deaths are due to placental infections [3]. Congenital malaria is the second mode of transmission of malaria after vector transmission. Infections of the placenta are very common in malaria-endemic countries, particularly in primiparous women [5]. About 20.2% of pregnant women have placentas infected [3, 6] by *Plasmodium falciparum* especially. This infection is a major cause of anaemia in pregnant women [7, 8]. In foetus, this placental sequestration of parasitized red blood cells alters the integrity of the placenta, reduces the transport of oxygen and nutrients, resulting in low birth weight [3]. This foetal hypotrophy can reach 170 g [6]. Newborns are twice likely to have low birth weight in the presence of placental infection [9]. Placental malaria can also cause premature birth [10] and even death of the mother and/or child. 5.7% of the deaths in Africa were due to placental infections during pregnancy [9]. Children should normally be protected by passive transmission of maternal immunoglobulin's, foetal haemoglobin, and colostrum [4]. However, it happens that children are born infected with *Plasmodium*. This infection can progress to clinical malaria known as congenital malaria disease.

The clinical manifestations of congenital malaria aren't most often specific [11]. The main clinical manifestations are fever, anaemia, and splenomegaly [8]. Jaundice, vomiting, and diarrhoea are also reported [12]. Biologically, it is characterized by parasitaemia and thrombocytopenia [13]. Two major clinical forms of congenital malaria are defined: (1) Congenital malaria infection, which is a transitory parasitaemia of peripheral blood of a neonate or its cord, clinically asymptomatic, and which spontaneously disappears in 2-3 days; (2) congenital malaria disease, which results in persistent parasitaemia with clinical manifestations during the first seven days of life. It has a spontaneous evolution, often fatal. These two forms must to be distinguished from the neonatal malaria, which is due to a postnatal inoculation of the parasite by the bite of infected female anopheles.

Congenital malaria infection is not uncommon in sub-Saharan Africa, and its prevalence varies from one site to another depending on the intensity of transmission [14]. This prevalence ranges from 10.8% to 54.2% [8]. In Niger, congenital malaria is poorly documented. Only two articles were published on the subject in 2000 [15] and 2006 [16].

It is in this context that we conducted a cross-sectional, prospective study to calculate the prevalence of congenital malaria, describe its clinical signs, and analyze its associated factors at Issaka Gazobi Maternity of Niamey-Niger.

## 2. Methodology

### 2.1. Methodology

It is a cross-sectional and prospective study to calculate the prevalence of congenital malaria, describe its clinical manifestations, and identify risk factors.

### 2.2. Site and Period of the Study

The study was conducted at Issaka Gazobi Maternity of Niamey, which is the National Reference Center (CNR) for Reproductive Health (RH), in collaboration with the Malaria Unit of the Center for Medical and Health Research, (CERMES) of Niamey. This site is located in a mesoendemic malaria transmission zone. This transmission is seasonal. It lasts 3 to 4 months, from July to October. The main vectors of malaria are *Anopheles gambiae* and *Anopheles coluzzi*. *Plasmodium falciparum* is the main transmitted parasite [17]. The study was conducted for six-months, from June 1 to November 30, 2017.

### 2.3. Study Population

The study population consisted of 249 consecutive mother/newborn pairs.

#### 2.3.1. Inclusion Criteria

Were included in this study all term neonates, hospitalized at Issaka Gazobi Maternity neonal department and their mothers. They are 0 to 7 days old.

#### 2.3.2. Criteria of Non Inclusion

Were not included in this study, all newborns without a health record, those who received a blood transfusion, premature babies and those whose parents did not consent.

### 2.4. Sampling

We used the simple probabilistic method. The inclusion of newborns followed the pace of hospitalization. With an estimated prevalence of 20% congenital malaria, a 95% confidence interval, and an accuracy of 5%, the sample size was 246 subjects.

### 2.5. Collection of Data

Data were collected from an individual clinical record to trace the history of each patient. The variables collected on mothers are the identifier, the age, the origin of the patient, the number of prenatal consultations, the notion of malaria or a notion of treatment of malaria during pregnancy, the observance of intermittent preventive treatment (IPT), the use of a long-lasting insecticide-treated net (LLIN), and a biological examination to evaluate the plasmodial infection.

The variables collected on the newborn were gestational age, birth weight, delivery route, newborn Apgar at birth, amniotic fluid colour, general condition of newborns (temperature, heart rate, respiratory rate), convulsive number, thick blood smear (TBS), thin smear (TS), blood count, Protein C Reactive (CRP), thick blood smear and thin smear cord blood, parasite density, plasmodial species, evolution of clinical signs, thick control after treatment, duration of hospitalization, and finally the way out of newborns.

### 2.6. Diagnosis of Congenital Malaria

Microscopy, which is the WHO reference method for diagnosing malaria, was used. Each recruited newborn received a thick blood test (TBT) and thin smear (TS) of peripheral blood and umbilical cord blood by layer. If he is positive at birth, a TBT and TS control were performed at 48 hours.

Each recruited mother had received a thick blood test and thin smear of peripheral blood from her fingertips.

#### 2.6.1. Thick Drop and Thin Smear

The blades of each patient are identified with the number of his medical file. The preparation of thick blood test and thin smears was made in accordance with WHO protocol [18].

#### 2.6.2. Staining of the Blades

The dried slides were stained with May Grunwald Giemsa diluted 1/10 for 15 minutes. They are then rinsed and left in the open to dry.

#### 2.6.3. Reading Slides

The slides were read under an optical microscope at 100X magnification. The thick drop was used to search and count the parasite. The parasite density was calculated by counting the number of parasites in parallel with 200 leucocytes. The parasite density is determined by the following formula: PD = Number of parasite counted × 8000/40. The thin smear was used to identify the plasmodial species.

#### 2.6.4. Haematological et Biochemical Tests

All haematological and biochemical examinations were performed at Issaka Gazobi Maternity Biology Laboratory using an automatic device (Reference: MSLAB O7 Plus). The parameters measured are red blood cell count, white blood cell count, hematocrit, platelets, blood sugar, and CRP.

### 2.7. Ethical Consideration

This study was authorized by the Faculty of Health Sciences of the University of Niamey as part of its doctoral dissertation work for 2017-2018. The anonymous nature of the questionnaire makes it possible to guarantee the confidentiality of the information collected. The informed consent of all mothers was obtained before their inclusion.

### 2.8. Ethical Consideration

An entry mask was created using Epi Info 7.0 software. All data has been entered and processed with this software. Chi-square Pearson and Fisher tests were used to compare percentages. A threshold of significance of 5% was retained.

## 3. Results

### 3.1. Characteristics of the Population

#### 3.1.1. Characteristics of Mothers

Two hundred and forty-nine (249) mothers were included in the study. The mean age of mothers was 27 years (SD: 6.2, [12; 42]). 92.77% (240/249) of women came from the Urban Community of Niamey (UCN), and 7.23% (9/249) came from rural areas. 96.79% (241/249) had given birth to Issaka Gazobi Maternity, and 3.21% (8/249) had been referred.

#### 3.1.2. Characteristics of Newborns

Two hundred and forty-nine (249) newborns were also included in the study. The sex ratio of newborns was 0.9. They were all born to terms. The age of newborns at inclusion varies from zero to seven days. Their average weight was 2754 g (DS: 588; [1050; 4600]). Their average size was 47.23 cm (DS: 3.31; [34; 54]). The characteristics of the population are summarised in Table 1.

### 3.2. The Prevalence of Congenital Malaria

#### 3.2.1. Prevalence of Congenital Malaria Infestation

26.51% (66/249) of newborns had a positive tick drop. The mean parasite density was 101 P/*μ*l (DS: 47.3, [80; 320]). All infections (100%) were *Plasmodium falciparum*.

#### 3.2.2. Prevalence of Congenital Malaria Disease

Forty-eight hours after the first biological examination, the thick blood drop remained positive in 14.06% (35/249) of neonates with a parasite density of 108 P/*μ*l (SD: 32.6; [40; 200]), confirming a persistent infection two days after birth.

### 3.3. Clinical Features of Congenital Malaria

60% (21/35) of newborns had at least one general sign after delivery. 51.43% (18/35) of newborns had a temperature above 37.5°C. 42.86% (15/35) of newborns had an Apgar at eight [8]. 20% (7/35) of newborns had a Blantyre score of 4. 51.43% (18/35) of newborns did not have suction reflex. 2.86% of newborns (1/35) had cyanosis, and 8.57% (3/35) had pallor. 11.43% (4/35) had seizures. 20% (7/35) of newborns had coma. 8.57% (3/35) had jaundice, and 45.71% (16/35) had low birth weight.

97.14% (34/35) of neonates were treated with artemether-lumefantrin in oral suspension for three days and 2.86% (1/35) with injectable artesunate. All the thick blood smear of the treatment control was negative. There was no relapse after treatment. The average duration of treatment was three days. All newborns were cured, and no deaths were recorded.

### 3.4. Biological Characteristics of Congenital Malaria

The mean parasite density of neonates with congenital malaria disease was 108 P/*μ*l (SD: 32.6; [40; 200]). The average temperature was 37.6°C (DS = 0.5, [36.5, 39.2]). The mean heart rate was 155 beats per minute (SD = 10.69; [135; 180]). The mean respiration rate was 49 per minute (SD = 8.2; [34; 66]). The mean haemoglobin level was 14.8 g/dl (SD = 2.6, [7.7, 19.9]). The mean haematocrit was 40.7% (SD = 6.9, [21; 54]). The platelet average was 259.4 (DS = 93205, [88,000, 459,000]). The average number of white blood cells was 14057 (SD = 6955, [4300, 38,000]). The mean glucose level was 0.79 g/L (SD = 0.54, [0.26, 1.87]). The average of C-Reactive Protein (CRP) was 8.7 (DS = 6; [5; 24]).

### 3.5. Determinants of Transmission of Congenital Malaria

#### 3.5.1. Prenatal Consultations

95.18% of pregnant women had prenatal consultations. Twenty-six point ten percent (65/249) of the mothers had three antenatal visits during pregnancy. 24.10% (60/249) completed four prenatal consultations, and 4.82% (4/35) did not attend prenatal consultations. Prenatal consultation is not a protective factor against congenital malaria. 88.57% (31/35) of neonates from prenatal mothers have had congenital malaria compared to 11.43% (4/35) of nonattenders. There is no difference in congenital malaria between the groups of women who consult and those who do not consult.

#### 3.5.2. Intermittent Preventive Treatment (IPT)

Twenty-seven point thirty-one percent (68/249) of pregnant women received no dose of sulfadoxine-pyrimethamine (SP) during pregnancy. 24.9% (62/249) received one and two IPT, respectively. 22.09% (55/249) received three intermittent preventive treatments. Newborns of mothers who do not take IPT are more exposed to congenital malaria. In fact, 63% (22/35) of newborns of mothers who did not take IPT made congenital malaria compared to 37% (13/35). Children of mothers who did three IPTs had less congenital malaria (5.71%) than mothers who did two IPTs (28.57%) and those who did not receive IPT (37.14%).

#### 3.5.3. Use of Insecticide-Treated Mosquito Nets

Sixty-eight point sixty-seven percent (171/249) of women did not sleep under a long-lasting insecticide-treated bed net. Newborns of women who use LLINs have had less congenital malaria than women who do not use it. There is a statistically significant difference in congenital malaria between the two groups (*p* = 0.04). Long-lasting insecticidal mosquito net is a protective factor against congenital malaria.

#### 3.5.4. Notion of Fever or Malaria during Pregnancy

Twenty-four point one percent (60/249) of women had fever during pregnancy. Newborns of women who have had a fever during pregnancy are half likely to be exposed to congenital malaria.

### 3.6. Analysis of Factors Associated with Congenital Malaria

Table 2 summarizes the analysis of all the factors that could be associated with congenital malaria, in particular prenatal consultation, use of insecticide-treated nets (IDNs), intermittent preventive treatment (IPT), and notion of malaria or fever during pregnancy.

## 4. Discussion

This cross-sectional and prospective study, conducted from 1^th^ of June to 30^th^ of November 2017 at the Neonatology Department of Issaka Gazobi Maternity in Niamey, describes the epidemiological, clinical, and biological characteristics of congenital malaria, analyses the factors of its transmission, and finally proposes preventative measures.

The study was conducted on 249 pairs of mothers and newborns. The prevalence of congenital malaria infection, objectified by a positive thick blood slide, was 26.51% (66/249) with an average parasite density of 101 P/*μ*l (SD: 47.3; [80; 320]). Congenital malaria infection is common in sub-Saharan Africa [14]. Its prevalence varies from 10.8% to 54.2% depending on the intensity of transmission [8], the collection period, and the biological confirmation method. The first study conducted in Niger in 2000 gave a prevalence of 13.3% [15]. It was 19% in Togo with a parasite density ranging from 360 to 870 P/*μ*L [19], 10% in Burkina [20], 2.2% in Ghana [21], 0.64% in Brazzaville [22], 0.03% in Mali [23], and 0% in Burundi [24]. Menendez and Mayor explain this wide variation in the prevalence of congenital malaria infection by: (I) the difference in the definition of congenital malaria, (II) the level of maternal immunity, (III) the type of blood examined (peripheral blood or the umbilical cord blood of the newborn), (IV) the expertise of microscopists, (V) the method of biological confirmation (microscopy, rapid diagnostic test, or chain polymerization reaction), and finally, (VII) the intensity of malaria transmission from the site [25]. In utero transmission of *Plasmodium* from mother to child by the placenta is accidental because the foetuses are normally protected by the maternal antibodies, foetal haemoglobin, and colostrum that the newborn receives [4].

The prevalence of congenital malaria disease is mostly low compared to that of congenital malaria infection [20]. On the other hand, the parasite density is most often high. It was 14.06% (35/249) with a parasite density of 108 P/*μ*l (SD: 32.6, [40; 200] in Niger, a prevalence of 24.4% in Burkina Faso [2], 1.7% in Togo with a parasite density ranging from 700 to 3000 P/*μ*L [19], and 1.05% in Senegal [26].

The incriminated plasmodium species are most often *Plasmodium falciparum*. All infections were *Plasmodium falciparum* at the Issaka Gazobi Maternity in Niamey, as in Ghana [21]. *Plasmodium vivax* predominates (92.50%) in Tanzania [12], and finally, *P. falciparum* and *P. ovale* were found in Mali [23].

Clinical manifestations of congenital malaria are most often not specific [11]. The main signs are fever, anaemia, and splenomegaly [8]. Jaundice, vomiting, and diarrhoea are also reported [12]. Biologically, it is characterized by parasitaemia [27] and thrombocytopenia [13]. In this series, hyperthermia, loss of sucking reflex, convulsion, coma, and pallor, and especially low birth weight are the most prominent signs. In fact, 45.71% of newborns had a low birth weight. The prognosis of congenital malaria is most often favorable. The treatment instituted was an artemisinin-based combination therapy suspension, especially artemether-lumefantrin. Prior to 2005, quinine was prescribed. However, since the AQUAMAT [28] and SEAQUAMAT [29] studies, artemether and artesunate have been prescribed [8]. No deaths were recorded in this study compared with 25% in Togo [19].

Only the nonuse of long-lasting insecticidal nets was significantly associated with congenital malaria (*p* = 0.04). Newborns of mothers with fever during pregnancy are at least twice at risk of congenital malaria. Fever is a bad prognostic factor for congenital malaria. Intermittent preventive treatment tends to show a protective effect against congenital malaria: Only thirty-seven percent of newborns in IPT mothers have had congenital malaria compared to 63% of those who do not. The effect of IPT also increases with the dose. Only 5.71% of newborns of mothers who did three IPTs had congenital malaria compared to 28% of those who had one dose of IPT.

Several recommendations are made to diagnose and treat congenital malaria. Francisca et al. believes that in malaria-endemic areas, any newborn with a sepsis chart should be investigated for possible congenital malaria. This recommendation will be very strong if the mother has not correctly taken antimalaria prophylaxis [30]. Other alternatives are also possible such as microscopic examination of placentas. For the prevention of congenital malaria, in the stable malaria transmission zone, the use of long-lasting insecticide-treated bed nets [31] and intermittent preventive treatment for pregnant women with sulfadoxine-pyrimethamine are the mains recommendations of WHO [25, 32].

The main limitation of this study is the lack of a serological assessment to confirm the exposure and use of PCR to highlight submicroscopic infections.

## 5. Conclusion

The frequency of congenital malaria infection is very high in Niamey where transmission is permanent with a strong recrudescence in the rainy season. On the other hand, the prevalence of congenital malaria disease is lower. The main risk factor is the lack of use of mosquito nets. Intermittent preventive treatment and distribution of long-lasting insecticide-treated mosquito nets should be provided to all pregnant women to prevent congenital malaria.

## Figures and Tables

**Table 1:** Characteristics of the population.

Characteristics	Mothers	Newborns	SD	IC95
Number included	249	249	_	_
Mean âge	27 years	_	6.2	[12; 42]
Sex ration	_	0.9	_	_
Average weight (g)	_	2754	588	[1050; 4600]
Average size (cm)	_	47.23	3.31	[34; 54]
Congénital malaria (%)	_	26.51	_	_
Parasite density (P/ùl)	_	101	47.3	[80; 320]
Congenital malaria disease (%)	_	14.06	_	_
Parasite density (P/ùl)	_	108	32.6	[40; 200]

**Table 2:** Analysis of factors associated with congenital malaria.

Variable	Number	Congenital Malaria (%) (12)00(17)00(26)00	Rapport of prevalence	*p* value
Prenatal consultation				
Yes	31	88.57%	7	NS
No	4	11.43%		
Usage of long-lasting insecticide-treated net				
Yes	12	34.29%	1.17	0.04
No	23	65.71%		
Intermittent preventive treatment with sulfadoxine-pyrimethamine				
Yes	13	37%	1.9	NS
No	22	63%		
0 IPT	13	37.14%		
1 IPT	10	28.57%		
2 IPT	10	28.57%		
3 IPT	2	5.71%		
Notion of fever and malaria treatment during pregnancy				
Yes	8	23%	2.1	0.5
No	27	77%		

## Data Availability

The excel file data used to support the findings of this study are available from the corresponding author upon request.
